# Development of an application for management of drug holidays in perioperative periods

**DOI:** 10.1097/MD.0000000000020142

**Published:** 2020-05-08

**Authors:** Sakiko Kimura, Akiko Emoto, Mariko Yoshimura, Kota Arimizu, Tomoko Kamura, Rintaro Sogawa, Kikumi Mizuta, Yasuhiro Tagomori, Masahiro Natsuaki, Masataka Kajiwara, Nanae Tsuruoka, Yusuke Yakushiji, Yoshinori Tanigawa, Chihiro Takamatsu, Atsushi Danjo, Keiji Kamohara, Naomi Hirakawa, Yoshiro Sakaguchi, Mitsuru Noguchi, Hirokazu Noshiro, Atsushi Kawaguchi, Eisaburo Sueoka, Yutaka Narisawa, Shinya Kimura

**Affiliations:** a Safety Management Section, Saga University Hospital; b Dep. of Pharmacy, Saga University Hospital; c Clinical Research Center, Saga University Hospital; d Department of Nursing, Saga University Hospital; e Div. of Cardiovascular Medicine, Dep. of Internal Medicine, Faculty of Medicine, Saga University; f Div. of Gastrointestinal Endoscopy, Dep. of Internal Medicine, Faculty of Medicine, Saga University; g Division of Neurology, Department of Internal Medicine, Faculty of Medicine, Saga University; h Surgical Center, Saga University Hospital; i Dep. of Oral and Maxillofacial Surgery, Faculty of Medicine, Saga University; j Dep. of Thoracic and Cardiovascular Surgery, Faculty of Medicine, Saga University; k Dep. of Anesthesiology and Critical Care Medicines, Faculty of Medicine, Saga University; l Dep. of Urology, Faculty of Medicine, Saga University; m Dep. of Surgery, Faculty of Medicine, Saga University; n Center for Comprehensive Community Medicine, Faculty of Medicine, Saga University; o Dep. of Clinical Laboratory Medicine, Faculty of Medicine, Saga University; p Div. of Dermatology, Dep. of Internal Medicine, Faculty of Medicine, Saga University; q Division of Hematology, Respiratory Medicine and Oncology, Department of Internal Medicine, Faculty of Medicine, Saga University, Japan.

**Keywords:** antithrombotic drug, application, perioperative drug management, SAMPOP

## Abstract

Before surgery and other invasive treatments, decisions must be made on whether to discontinue drugs and provide appropriate drug holidays especially for antithrombotic drugs, and this is made difficult by the large number of available drugs and associated guidelines. We have therefore developed an online application for perioperative drug discontinuation and resumption management, named Saga Application for Management of Drug Holidays in PeriOperative Periods (SAMPOP).

Multidisciplinary medical staff at Saga University Hospital (SUH) worked together to build an evidence-based Perioperative Drug Discontinuation Management Database (PDDMD) and developed the user-friendly SAMPOP online application via preliminary verification at SUH. From September 2018 to February 2020, 420 medical staff at SUH, including physicians, nurses, and pharmacists, installed and tested SAMPOP.

Rate per surgical procedure for forgetting to discontinue antithrombotic drugs preoperatively decreased from 0.18% to 0.09% as of August 2019, 12 months after the introduction of SAMPOP (*P* = .1359). In addition, six months later, it decreased further to 0.03% as of February 2020 (*P* = .0436). Forgetting to resume antithrombotic drugs postoperatively decreased from 0.20% to 0.02% as of August 2019, 12 months after the introduction of SAMPOP (*P* = .0008). There was no case of forgetting to resume the medication in the last 6 months.

SAMPOP may be useful for management of drug holidays in the clinic and warrants further evaluation of its safety and efficacy.

## Abstract 2

Supplemental Digital Content is available in the text

## 1 Introduction

Failure to appropriately discontinuing antithrombotic drugs such as anticoagulants and antiplatelet agents before surgery, endoscopic treatment, intrathecal injection, or other invasive treatments can force the postponement of treatments and changes in the method of anesthesia. Furthermore, in unfortunate cases this may induce unexpected hemorrhage, but conversely, inappropriate discontinuation of antithrombotic drugs increases the risk of thrombosis. Numerous guidelines and approaches for drug holidays have been published on perioperative and other invasive treatments.^[1–20]^ However, as novel agents are introduced and as guidelines continue to change, it is a challenge for medical staff to remain up to date with the latest information. In addition, it is very difficult to unify drug management among diverse medical staff.

Therefore, in the present work, we attempted to develop an online application for perioperative antithrombotic drug discontinuation and resumption management, named Saga Application for Management of Drug Holidays in PeriOperative Periods (SAMPOP), based on available evidence and best practice. We subsequently investigated the efficacy and safety of SAMPOP by comparing pre- and post-induction rates per surgical procedure of forgetting to discontinue antithrombotic drugs preoperatively and of forgetting to resume antithrombotic drugs postoperatively, with and without SAMPOP. We also investigated the incidence of perioperative hemorrhagic and thrombotic events. After the SAMPOP pilot study, 169 patients who were taking antithrombotic drugs and admitted via the Medical Support Center to Saga University Hospital (SUH) for operations were evaluated retrospectively using SAMPOP, and safety and efficacy were explored in detail.

## 2 Methods

### 2.1 Perioperative drug discontinuation management database (PDDMD)

The Multidisciplinary Hemostasis/Thrombosis Management Team, Drug Information Office, and Medical Safety Management Office of SUH coordinately built an evidence-based PDDMD (Table 1) based on extensive guidelines and published approaches.^[1–20]^ We initially classified and evaluated hemorrhagic risks in anesthesia, surgery, and gastrointestinal endoscopy (Supplementary Table 1),^[1,6,8,19,20]^ as well as thrombotic risks in coronary artery diseases, stroke, atrial fibrillation, and venous thromboembolism (Supplementary Table 2).^[4,6,7,9,12]^ We subsequently analyzed the drug information and built the PDDMD (Table 1).^[1,2,7,8,10,11,13,14]^

**Table 1:**
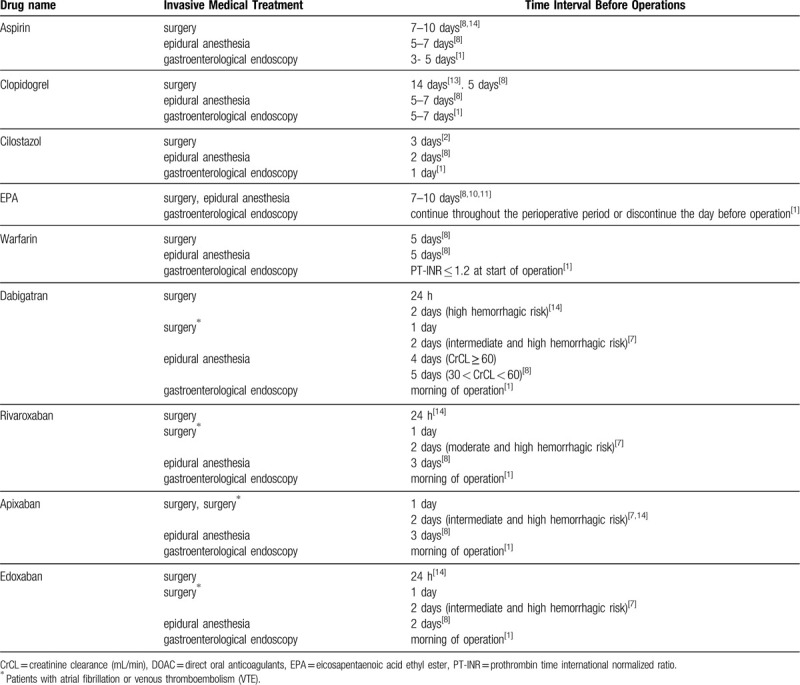
Perioperative Drug Discontinuation Management Database (PDDMD).

### 2.2 Programming of SAMPOP

**Figure 1:**
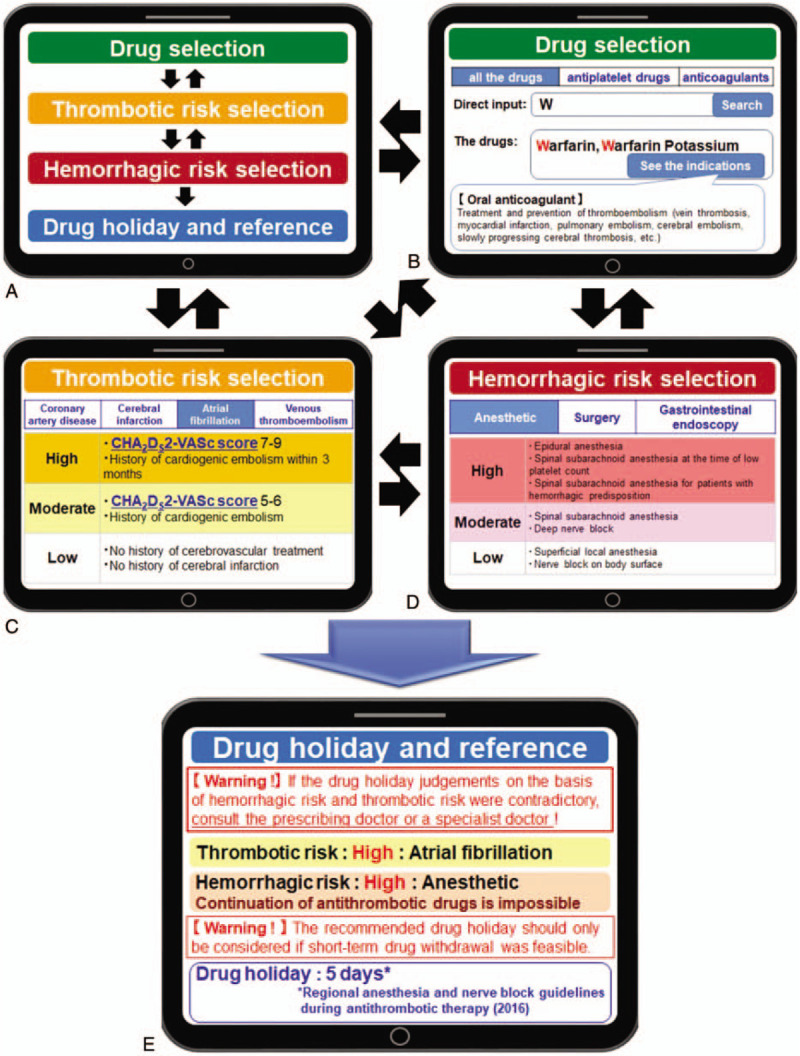
The SAMPOP operating screen (English version). (A) In the first screen, 3 tags (drug, thrombotic risk, and hemorrhagic risk) are displayed and can be selected by the user. (B) All drugs can be searched for as strings or selected from a list of antithrombotic drugs, including anticoagulants and antiplatelet drugs. (C) SAMPOP is programmed to enable users to select whether the subject is at high, moderate, or low risk of coronary artery disease, cerebral infarction, atrial fibrillation, or venous thromboembolism under treatment with antithrombotic drugs. (D) SAMPOP is programmed to enable users to select whether the subject is at high, moderate, or low risk of scheduled anesthesia, surgery, other invasive procedures, or gastrointestinal endoscopy. (E) After answering all tags, in the final page, drug holidays and reference sources are displayed.

To determine appropriate drug holidays, SAMPOP performs three steps: drug selection, hemorrhagic risk, and thrombotic risk (Fig. 1). The programming languages used to develop SAMPOP were Hypertext Preprocessor (PHP Ver.7.2) and SQLite Ver.3 for the database. Programs for SAMPOP were designed to enable easy data maintenance by both computer programmers and ordinary supervisory medical staff. A secure web server was used for hosting, and to ensure that updated data were immediately available to SAMPOP users, we enabled online access via an internet browser with an online display for mobile devices, including tablet computers and smart phones.

### 2.3 Evaluation of SAMPOP

The number of medical staff per month who installed SAMPOP and the total number of accesses to the software in each department of SUH were counted via the internet. The efficacy and safety of SAMPOP were retrospectively investigated by comparing the rates per surgical procedure of forgetting to discontinue antithrombotic agents preoperatively and rates of forgetting to resume antithrombotic drugs postoperatively over 3 periods; pre- (September 2016 to August 2017), post- (September 2018 to August 2019) and post- (September 2019 to February 2020) induction of SAMPOP.

Before admission for surgery and other invasive treatments, pharmacists in the medical support center of SUH checked which medicines each patient was taking, and if a patient was taking antithrombotic drugs, the pharmacist consulted his/her attending physician to determine whether the patient should have a drug holiday during the perioperative period. The physician was then free to decide whether SAMPOP should be used to determine drug holidays.

Electronic medical records were used to identify

(1) patient age,
(2) the department to which each patient was referred,
(3) the reason for taking antithrombotic drugs,
(4) the scheduled method of anesthesia and surgical procedure,
(5) the physician's orders for drug holidays,
(6) the physician's orders to resume drugs postoperatively,
(7) perioperative hemorrhagic events requiring blood transfusion,
(8) thrombotic events requiring treatment, and
(9) the actual date of resumption of any drug.

In addition, the study examined whether the attending doctors who forgot to discontinue or resume antithrombotic drugs perioperatively had installed SMPOP or not, as well as whether attending doctors who had installed SAMPOP appropriately accessed SAMPOP perioperatively; 1 month before the scheduled invasive treatments in the case of forgetting to discontinue and one week before and after the invasive treatments in the case of forgetting to resume antithrombotic drugs.

After the pilot induction of SAMPOP (September 2018 to March 2019), 169 patients who were taking antithrombotic drugs were admitted to SUH via the Medical Support Center underwent operations. Patient characteristics are summarized in Table 2. For all 169 patients, the answers given by SAMPOP were retrospectively evaluated (Fig. 1E), especially the thrombotic and hemorrhagic risks for each patient based on their electronic medical records, and the drug holidays recommended by SAMPOP were recorded. The present retrospective study was conducted in accordance with the Declaration of Helsinki. Approval of the study protocols was obtained from the SUH Clinical Research Ethics Committee, and informed consent was obtained as opt-out (2018-10-rapid-01).

**Table 2:**
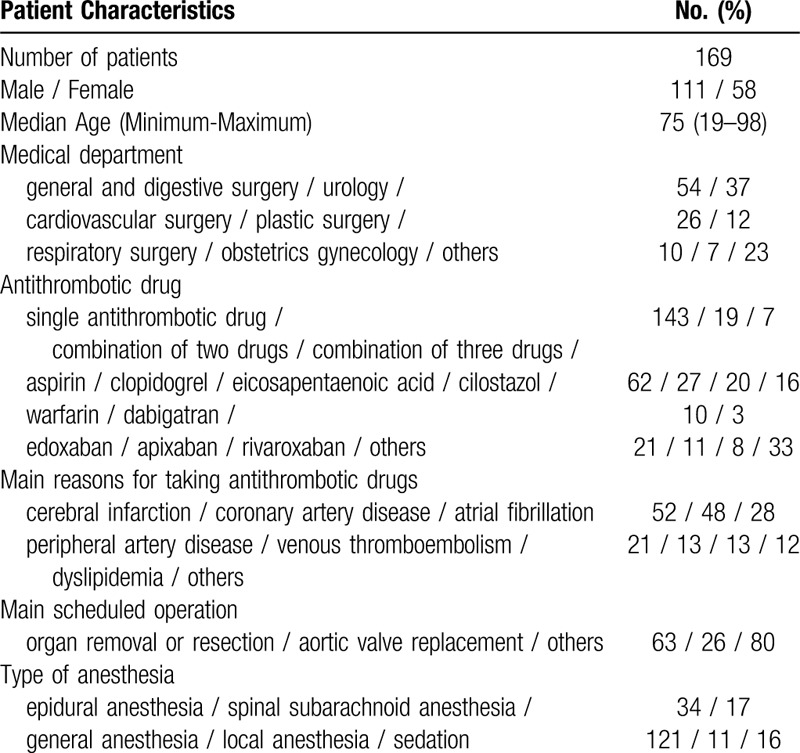
Patient characteristics.

### 2.4 Statistical analysis

The JMP (14.2.0) statistical application was used for analysis, with incidences compared using Fisher exact tests; differences between mean values determined by one-tailed *t* tests; and 95% confidence intervals for means calculated from t-distributions (*P* < .05 was considered statistically significant).

## 3 Results

### 3.1 Structure of SAMPOP

Figure 1 shows the English version of the operating procedure of SAMPOP. At present, only SAMPOP with the Japanese operating procedure is in actual use (Supplementary Figure 1); however, if the English version is required, it can be provided rapidly upon reasonable request. A SAMPOP user first selects one of 3 tags,

(i) Drug,
(ii) Thrombotic Risk, and
(iii) Hemorrhagic Risk, which are displayed on the first screen via a touch panel (Fig. 1A).

After answering the questions that appear on the next page (Fig. 1B–D), the process is repeated for the other two tags. Tags are selected in a random order, and when the process is complete, recommended drug holidays and source references are displayed (Fig. 1E).

A master record was created in SAMPOP for all 74 drugs containing 25 active ingredients listed in the PDDMD, enabling all “drugs” to be searched for as strings or selected from a list of antithrombotic drugs, including anticoagulants and antiplatelet drugs. The program also enables users to check indications for each drug (Fig. 1B). SAMPOP is also programmed to enable users to select whether the patient is at high, moderate, or low thrombotic risk from each tag, based on each patient's history of coronary artery disease, cerebral infarction, atrial fibrillation, venous thromboembolism, and other ailments. For atrial fibrillation, SAMPOP also calculates the CHA_2_DS_2_–VASc risk stratification scores^[21]^ linked to thrombotic risk category (Fig. 1C). SAMPOP is also programmed to enable users to select whether a patient is at high, moderate, or low hemorrhagic risk from scheduled anesthetic, surgery, or gastrointestinal endoscopy (Fig. 1D). It takes only a few seconds for the application to select each tag, hence the time required from launching SAMPOP to displaying drug holidays and source references is less than a minute. When the above 3 steps are complete, drug holidays and source references are displayed. When drug holiday judgments based on hemorrhagic and thrombotic risk are contradictory, SAMPOP recommends that the user consult a specialist (Fig. 1E).

### 3.2 Evaluation of SAMPOP

**Figure 2:**
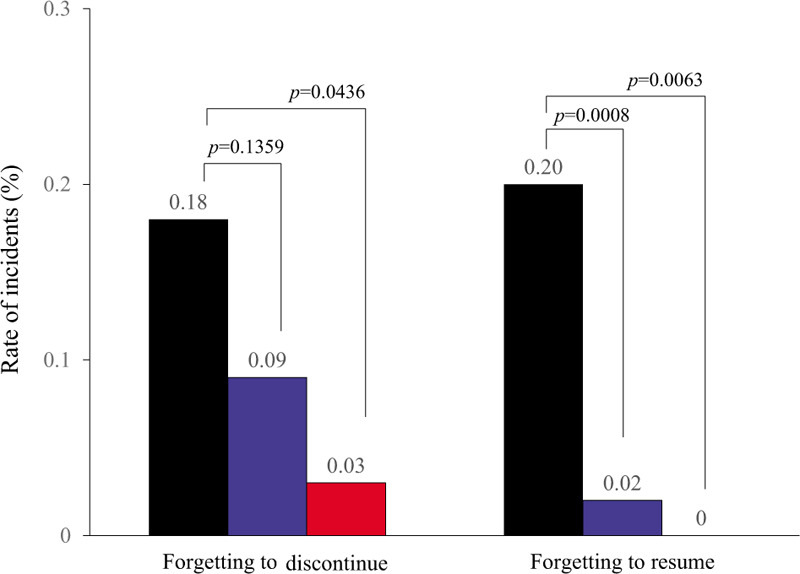
Rates of forgetting to discontinue antithrombotic drugs preoperatively and of forgetting to resume them postoperatively. The rates per surgical procedure of forgetting to discontinue antithrombotic drugs and to resume antithrombotic drugs postoperatively during one year pre-SAMPOP induction (September 2016–August 2017; n = 6652) (black bar), and post-SAMPOP induction: September 2018–August 2019, n = 6894 (blue bar); September 2019–February 2020, n = 3486 (red bar).

One month after the introduction of SAMPOP (September 2018), the number of medical staff who installed SAMPOP increased immediately. After the second month of induction, it became almost plateau (13.5 persons on average with a distribution range of one to 41 persons per month). From April 2019 to June 2019, the number of installations was a little bit higher than in other months, because the Japanese business year starts in April and most hiring of new staff begins in this month (Supplementary Figure 2). At present (February 2020), 39.2% of all medical staff at SUH have installed SAMPOP. The numbers of operations performed in SUH 1 year pre-introduction (September 2016–August 2017), 1 year post-introduction (September 2018–August 2019) of SAMPOP, and in the last 6 months (September 2019–February 2020) were 6652, 6894, and 3486, respectively. Although it is unclear whether all patients were evaluated by SAMPOP after pilot induction, the rate per surgical procedure for forgetting to discontinue antithrombotic drugs preoperatively decreased from 0.18% to 0.09% as of August 2019, 12 months after the introduction of SAMPOP (*P* = .1359). In addition, it decreased further to 0.03% in the last 6 months (*P* = .0436). Forgetting to resume antithrombotic drugs postoperatively decreased from 0.20% to 0.02% as of August 2019, 12 months after the introduction of SAMPOP (*P* = .0008). Intriguingly, there was no case of forgetting to resume medication in the last 6 months (Fig. 2).

Among attending doctors who had associated incidents of forgetting to discontinue and/or resume antithrombotic drugs, 75.0% had not installed SAMPOP when the incidents occurred. Interestingly, when doctors had forgotten to discontinue antithrombotic drugs, none had accessed SAMPOP during the perioperative period.

Cases of forgetting to discontinue a drug that should have been discontinued preoperatively were discovered by pharmacists working at the Medical Support Center. These cases were reported as soon as possible to the attending physicians, who then took the necessary action prior to surgery to ensure that patients did not have an operation while still taking an antithrombotic drug. Cases of forgetting to resume a drug were identified by ward pharmacists, who told the attending physicians, and then patients immediately resumed taking the drug.

### 3.3 Discrepancies between physicians’ orders and recommendations by SAMPOP

The time to postoperative resumption of treatment ordered by physicians tended to be longer than the period recommended by SAMPOP in the 169 patients (Table 2). Recommendations by SAMPOP, including the percentage giving a clear answer about drug holidays and orders by each physician, are summarized in Table 3. Since some patients took more than one antithrombotic drug, 178 antithrombotic drugs were included in the 169 patient study. Among these 178 drugs, SAMPOP gave clear answers for drug holidays in 43.3% of cases. In other words, in 56.7% of cases, SAMPOP recommendations alone were insufficient and required user consultations with specialists to arrive at a definitive decision.

**Table 3:**
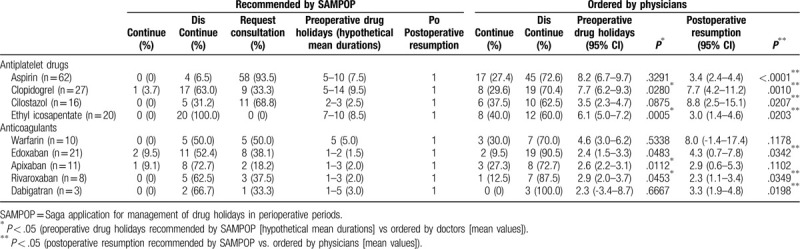
Drug holidays recommended by SAMPOP and ordered by physicians.

Regarding aspirin (n = 62), SAMPOP gave clear answers for 4 patients in whom it recommended discontinuation, with 7 to 10 drug holidays and 1 day postoperative resumption (Table 3). Among 62 patients, 45 (72.6%) discontinued aspirin with 8.2 drug holidays, comparable to the recommendations by SAMPOP. However, the other 27.4% of patients did not discontinue aspirin, and actual postoperative resumption of aspirin was 3.4 days, which was much longer than SAMPOP recommendations (*P* < .0001). In patients treated with clopidogrel, ethyl icosapenate, apixaban, or rivaroxaban, the actual drug holidays ordered by physicians were longer than the hypothetical mean durations presented by SAMPOP. In most cases, except in patients who were treated with warfarin or apixaban, the actual number of days to resume antithrombotic drugs was much longer than that recommended by SAMPOP in most cases (Table 3).

### 3.4 Occurrence of hemorrhagic and thrombotic events

Sixteen patients had perioperative hemorrhagic events that required blood transfusion, and there were 2 postoperative thrombotic events that required treatment (Table 4). However, in one out of 16 cases of hemorrhagic events, the actual preoperative drug holidays ordered by physicians were shorter than those recommended by SAMPOP, and in 1 case, the physician had reasons why the patient could not discontinue antithrombotic drugs earlier. Additionally, in two cases of thrombotic events, the time to resume antithrombotic drugs was longer than that recommended by SAMPOP, but due to surgical procedures and characteristics, the delay in resumption of antithrombotic drugs was judged to be inevitable.

**Table 4:**
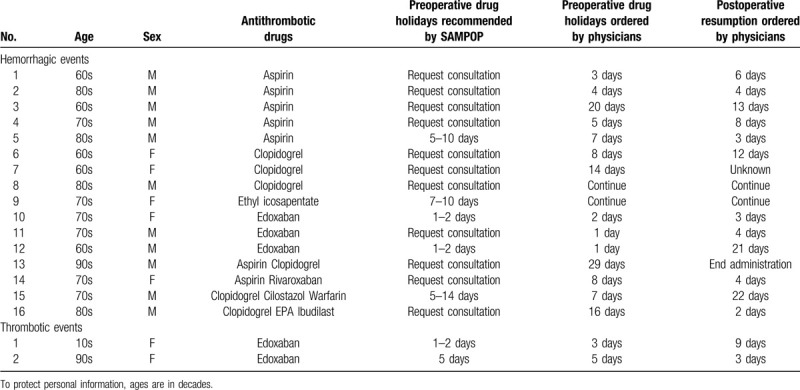
Occurrence of hemorrhagic and thrombotic events.

## 4 Discussion

A proper evidence-based assessment of both thrombotic and hemorrhagic risk is required for perioperative drug management of patients.^[22–24]^ Accumulating evidence indicates that drug management in perioperative periods has become more time- and labor-intensive. The SAMPOP developed herein makes it easier for the user to classify and assess whether or not a drug should be discontinued or continued based on available evidence (i.e., PDDMD; Table 1), depending on the specific drug, the hemorrhagic risk during surgery or other treatments, and the thrombotic risk based on patient condition (Fig. 1). Effective use of SAMPOP could prevent unnecessary drug treatment, unnecessary admission, or investigations due to delayed surgery, as well as complications due to inappropriate drug use, thereby contributing to medical safety and reducing medical costs.

To manage drugs appropriately, diverse medical staff, including physicians, nurses, and pharmacists, must work cooperatively. In addition, regional core hospitals, such as university hospitals, increasingly collaborate with local primary care institutions (hospitals and clinics). Thus, SAMPOP is likely to prove useful not only for medical staff in core hospitals but also in local primary care institutions, facilitating closer collaboration. After sufficient verification in SUH, we intend to distribute SAMPOP to local primary care institutions for use free of charge.

Before rolling out SAMPOP for use by the wider medical community, verification of safety and efficacy is mandatory. Herein, SAMPOP was preliminarily introduced and used for a limited test period in SUH, and although statistically inadequate, the rates of forgetting to discontinue antithrombotic drugs preoperatively and to resume them postoperatively were both decreased (Fig. 2). Because it was very difficult to determine whether SAMPOP was used for all patients who had surgeries or invasive treatments after the introduction of SAMPOP, the data presented in this paper do not fully demonstrate whether the observed improvement in adherence to drug holidays was due to the introduction of SAMPOP. For instance, some of the improvements (Fig. 2) might have been due to regulatory or policy changes regarding drug holidays introduced before or after the introduction of SAMPOP. However, we found no changes in such regulations or policies at SUH during the periods concerned. It was also unclear whether the increase in the number of SAMPOP installations led to a decrease in the number of incidents (Supplementary Figure 2, Fig. 2). However, it is certainly possible that the increase in the number of SAMPOP installations at least greatly contributed to awareness among medical staff at SUH about the necessity for drug holidays. In addition, as for the number of accesses by each clinical department, the largest number of accesses was by cardiologists who had received consultations from other attending doctors (Supplementary Table 3). Of the attending doctors who caused incidents, 75.0% had not installed SAMPOP. The percentage of appropriate accesses to SAMPOP in the both cases of forgetting to discontinue and resume antithrombotic drugs preoperatively was 0%. Taken together, these results demonstrate that SAMPOP has the potential to reduce the number of incidents of non-adherence to perioperative drug management policy.

Furthermore, the present study indicated that the physicians working at SUH tended to order shorter drug holidays for certain drugs such as clopidogrel and ethyl icosapenate, and longer drug holidays for others such as cilostazol, edoxaban, apixaban, and ribaroxaban, as well as a longer time to resume most antithrombotic drugs except for warfarin and apixaban, which were recommended by SAMPOP (Table 3). There may be several reasons why shorter drug holidays were ordered by physicians. One reason is the wide range of drug holidays described in the publications and guidelines that were used for evidence. For example, one guideline recommended a preoperative drug holiday of 5 to 7 days for clopidogrel.^[1,8]^ Meanwhile, the clopidogrel package insert states that its use should be discontinued at least 14 days preoperatively.^[13]^ When important publications recommend drug holidays of different periods, SAMPOP attempts to display all the recommended periods and their sources. To achieve this, regular updates are indispensable. SAMPOP is advantageous here because it can be easily updated by anyone responsible.

In addition to differences between guidelines and package inserts, in clinical practice, many physicians may order drug holidays for some drugs that differ from those recommended by SAMPOP because they may prioritize the individual characteristics of a patient, or the hemorrhagic risk of the operation or procedure, when making their decision. In practice, SAMPOP could not give clear answers in 56.8% of cases (Table 3). It is difficult to accurately reflect differences among individual patients using SAMPOP at present. When drug holidays that are determined based on hemorrhagic and thrombotic risk are contradictory, the current version of SAMPOP may need the assistance of specialists. However, in future, we intend to utilize artificial intelligence to solve this problem.

We found that 16 patients had perioperative hemorrhagic events that required blood transfusion and two postoperative thrombotic events that required treatment (Table 4). Although the causal relationships between drug holidays and hemorrhagic events were unknown, and most causes could be attributed to differences in individual status, the limitation of the current SAMPOP version is due to insufficient information about individual status. In addition, it was difficult for SAMPOP to give a clear answer in cases where novel surgical techniques, such as robot-assisted surgery, were performed. Evidence related to perioperative hemorrhagic and/or thrombotic events for these novel techniques remains limited.

Although SAMPOP currently focuses on antithrombotic drugs, we intend to expand this to other drugs. We have already added one agent, ibrutinib, which inhibits Bruton tyrosine kinase to target chronic lymphocytic leukemia and sometimes causes serious hemorrhages.^[25,26]^ For ibrutinib, drug holidays of 3 to 7 days before and after surgery are recommended.^[27]^ The hemorrhagic mechanism of ibrutinib is not yet known, but studies suggest that Bruton tyrosine kinase inhibition by ibrutinib may block platelet activation and aggregation.^[28,29]^ In addition to ibrutinib, we will add other agents that act on other molecular targets such as those causing bleeding or thrombosis, even though the frequency may be low.^[30–32]^ Furthermore, not all drugs that must be discontinued preoperatively are antithrombotic drugs or novel molecular target agents, but they also include other agents such as metformin, which increases the risk of serious lactic acidosis and thrombosis.^[33–36]^ Therefore, we are planning to incorporate as many drugs as possible into SAMPOP.

Because the present study was organized retrospectively, evaluation of its safety and efficacy was limited. We are planning a “prospective multicenter randomized trial” to verify the safety and effectiveness of SAMPOP. Hospitals will be randomized blindly with respect to use or nonuse of SAMPOP at the beginning of the prospective study and the number of incidents in each hospital will be determined 1 year before and 1 year after initiation of the trial.

## 5 Conclusions

We have developed a novel application for perioperative drug management named SAMPOP, which has potential for improving patient safety in perioperative periods.

## Author contributions

SK, AK, and SK made substantial contributions to study conception, design, analysis, and data interpretation. SK, AE, MY, KM, YT, MN, MK, NT, YY, TY, CT, NH, YS, HN, MN, KK, AD, ES, and YN contributed to the PDDMD. SK, MY, TK, RS, MN, MK, NT, YY, TY, HN, MN, KK, AD, and YN enrolled patients and collected data. SK, AE, MY, and KA programmed SAMPOP. SK and AK performed statistical analysis. SK and SK wrote the manuscript, and all authors approved the final version.

## Supplementary Material

Supplemental Digital Content

## Supplementary Material

Supplemental Digital Content

## Supplementary Material

Supplemental Digital Content

## Supplementary Material

Supplemental Digital Content

## Supplementary Material

Supplemental Digital Content
