# Supplementary figures and images for: Development of an application for management of drug holidays in perioperative periods
Source: Medicine (Baltimore). 2020 May 8;99(19):e20142. doi: 10.1097/MD.0000000000020142 (PMC7220215; doi:10.1097/MD.0000000000020142)

Supplementary Figure 1. Japanese version of SAMPOP

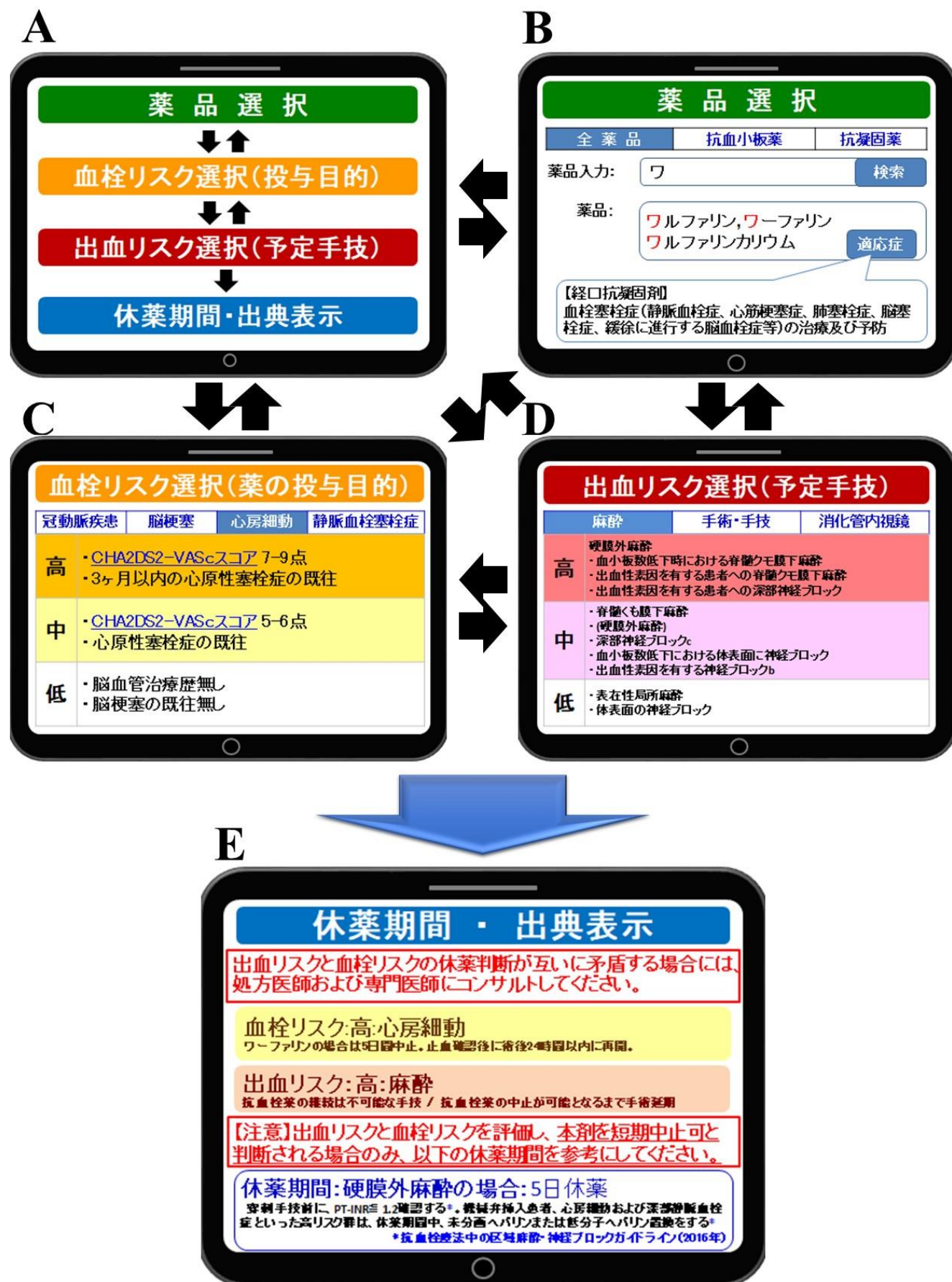

Supplement: Supplemental Digital Content [file medi-99-e20142-s001.pdf]
