# Supplementary material for: Development of an application for management of drug holidays in perioperative periods
Source: Medicine (Baltimore). 2020 May 8;99(19):e20142. doi: 10.1097/MD.0000000000020142 (PMC7220215; doi:10.1097/MD.0000000000020142)
Supplement: Supplemental Digital Content [file medi-99-e20142-s002.pdf]

Supplementary Figure 2 Number of SAMPOP installations and accesses to SAMPOP per month.

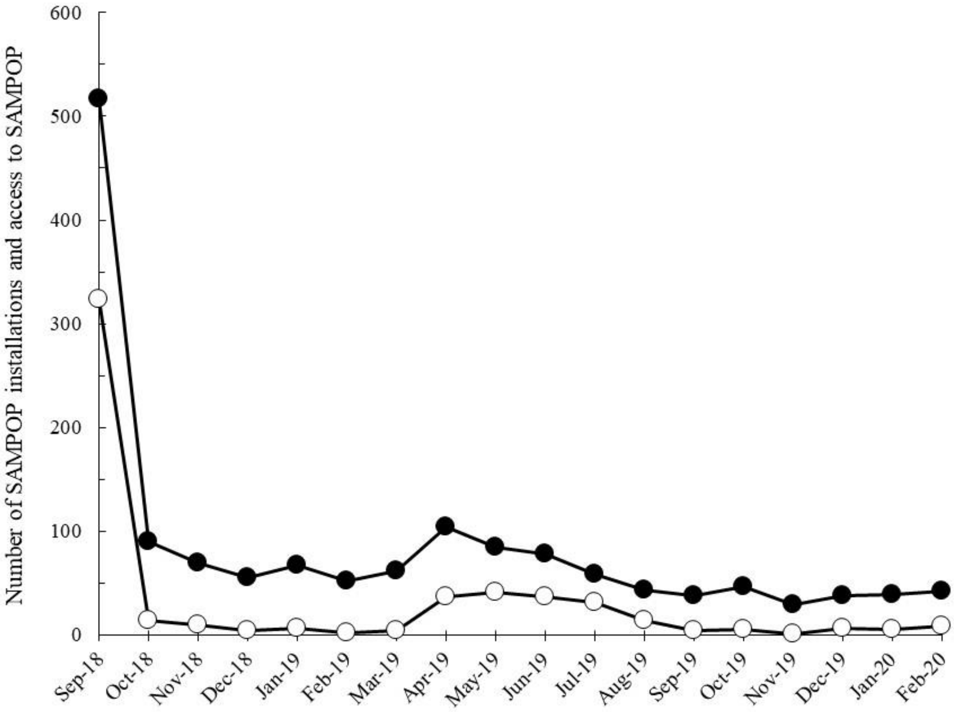

Open circle (○) and closed circle (●) indicate the total number of SAMPOP installations and accesses to SAMPOM per month.
