# Supplementary material for: Development of an application for management of drug holidays in perioperative periods
Source: Medicine (Baltimore). 2020 May 8;99(19):e20142. doi: 10.1097/MD.0000000000020142 (PMC7220215; doi:10.1097/MD.0000000000020142)
Supplement: Supplemental Digital Content [file medi-99-e20142-s003.docx]

**Supplementary Table 1. Database of hemorrhagic risk definition corresponding to invasive treatments**

| Main procedure |  | Hemorrhagic risk | Recommendation |
| --- | --- | --- | --- |
| Anesthesia^8^ | | | |
| ・epidural anesthesia  ・spinal anesthesia with thrombocytopenia or hemophiliac | | High | postpone surgery if antiplatelet therapy must be continued |
| ・spinal anesthesia  ・epidural anesthesia | | Moderate | No contraindications with aspirin alone |
| ・superficial local anesthesia | | Low | No contraindications with antiplatelet drugs |
| Surgery^6,8,19,20^ | | | |
| ・major invasive surgery (any procedure in which body cavity is entered, mesenchymal barrier is crossed, facial plane is opened, an organ is removed, or normal anatomy is altered)  ・lumber puncture, intrathecal injection | | High | postpone surgery if antiplatelet therapy must be continued |
| ・minor invasive surgery (any operative procedure in which only skin, mucous membranes, or superficial connective tissue are manipulated, as well as gastroscopy, colonoscopy, and similar)  ・bone marrow puncture or biopsy  ・central vein puncture of internal jugular and subclavian | | Moderate | No contraindications with aspirin alone |
| ・superficial local anesthesia  ・central vein puncture of femur | | Low | No contraindications with antiplatelet drugs |
| Gastrointestinal endoscopy^1^ | | | |
| Cases requiring professional consultation  ・EMR, ESD  ・gastrostomy | | High | postpone surgery if antiplatelet therapy must be continued |
| ・EMR, ESD  ・gastrostomy  ・endoscopy in case of taking multi-antithrombotic drugs | | Moderate | No contraindications with aspirin alone |
| ・endoscopy  ・ERCP | | Low | No contraindications with antiplatelet drugs |

EMR = endoscopic mucosal resection, ESD = endoscopic submucosal dissection, ERCP = endoscopic retrograde cholangiopancreatography
