# Supplementary material for: Development of an application for management of drug holidays in perioperative periods
Source: Medicine (Baltimore). 2020 May 8;99(19):e20142. doi: 10.1097/MD.0000000000020142 (PMC7220215; doi:10.1097/MD.0000000000020142)
Supplement: Supplemental Digital Content [file medi-99-e20142-s004.docx]

**Supplementary Table 2. Database of thrombotic risk definition corresponding to disease states**

| Disease | States | Thrombotic risk | Recommendations |
| --- | --- | --- | --- |
| CAD^4,6^ | | | |
| ・<1 month after coronary stent implantation or CABG | | High | ・elective surgery – postpone operation  ・non-elective surgery – continue aspirin and consider bridging therapy with heparin; resume other drugs within 24 h^a,b^; continue DAPT as long as possible^c^ |
| ・1−12 months after coronary stent implantation  ・1−6 months after CABG | | Moderate | ・elective surgery – postpone operation  ・non-elective surgery – continue aspirin alone and resume other drugs within 24 h |
| ・>12 months after coronary stent implantation  ・>6 months after CABG | | Low | ・in general, continue aspirin  ・discontinue aspirin and resume within 24 h^a^ |
| Stroke^9^ | | | |
| ・3 months after CAS  ・6 months after intracranial stenting  ・symptomatic carotid / intracranial major artery with over 50% stenosis  ・<6 months after TIA or stroke | | High | ・elective surgery – postpone operation  ・non-elective surgery – consider bridging therapy with heparin and continue aspirin or cilostazol at a minimum |
| ・asymptomatic carotid / intracranial major artery with over 50% stenosis  ・>6 months after stroke | | Moderate | ・in general, continue aspirin or cilostazol  ・discontinue for a short time and resume within 48 h |
| ・no medication for cerebral vessel  ・without previous stroke | | Low | ・discontinue for as long as possible and resume within 48 h |
| AF^7^ | | | |
| ・CHA_2_DS_2_-VASc score 7−9  ・<3 months after a history of cardiac cerebral embolism | | High | ・discontinue warfarin 5 days before surgery and start bridging therapy with heparin; resume within 24 h except for high hemorrhagic risk cases  ・discontinue DOAC 1 or 2 days^a,b^ before surgery |
| ・CHA_2_DS_2_-VASc score 5−6  ・history of cardiac cerebral embolism | | Moderate | ・discontinue warfarin 5 days before surgery and consider starting bridging therapy with heparin; resume within 24 h  ・discontinue DOAC 1 day or 2 days^a,b^ before surgery |
| ・no history of cerebral embolism | | Low | ・discontinue warfarin 5 days before surgery  ・discontinue DOAC 1 day or 2 days^a,b^ before surgery; bridging therapy with heparin is unnecessary |
| VTE^12^ | | | |
| ・<3 months after VTE  ・severe thrombophilia^12)^ | | High | ・discontinue warfarin 5 days before surgery and start bridging therapy with heparin; resume within 24 h except for high hemorrhagic risk cases  ・discontinue DOAC 1 day or 2 days^a,b^ before surgery |
| ・3−12 months after VTE  ・recurrent VTE  ・active cancer | | Moderate | ・discontinue warfarin 5 days before surgery and consider starting bridging therapy with heparin; resume within 24 h  ・discontinue DOAC 1 day or 2 days^a,b^ before surgery |
| >12 months after VTE | | Low | ・discontinue warfarin 5 days before surgery  ・discontinue DOAC 1 day or 2 days^a,b^ before surgery; bridging therapy with heparin is unnecessary |

(a) high hemorrhagic risk surgery; (b) moderate hemorrhagic risk surgery; (c) low hemorrhagic risk surgery. CHA_2_DS_2_-VASc, stroke risk scheme (congestive heart failure, hypertension, age ≥75 years, diabetes mellitus, stroke / transient ischemic attack, vascular disease, age 65−74 years, sex category).

CAD = coronary artery disease, AF = atrial fibrillation, VTE = venous thromboembolism, PCI = percutaneous coronary intervention with stent, CABG = coronary artery bypass grafting, DAPT = dual antiplatelet therapy, consisting of a combination of aspirin and P2Y_12_ receptor inhibitors, CAS = carotid artery stenting, TIA = transient ischemic attack, DOAC = direct oral anticoagulants
