# Supplementary material for: Development of an application for management of drug holidays in perioperative periods
Source: Medicine (Baltimore). 2020 May 8;99(19):e20142. doi: 10.1097/MD.0000000000020142 (PMC7220215; doi:10.1097/MD.0000000000020142)
Supplement: Supplemental Digital Content [file medi-99-e20142-s005.docx]

**Supplementary Table 3. Total number of accesses to SAMPOP after its introduction in each department of SUH**

| Occupation | Accesses  (n=596) |
| --- | --- |
| Medical Doctor  Div. of Cardiovascular Medicine  Dep. of Urology  Dep. of Anesthesiology  Div. of Dermatology  Div. of Hematology, Respiratory Medicine and Oncology  Dep. of Thoracic and Cardiovascular Surgery  Dep. of Obstetrics and Gynecology  Dep. of Oral and Maxillofacial Surgery  Dep. of Surgery  Div. of Neurology  Dep. of Otolaryngology - Head & Neck Surgery  Dep. of Orthopedic Surgery  Dep. of General Medicine  Div. of Metabolism and Endocrinology  Dep. of Radiology  Div. of Nephrology  Others | **118**  55  46  39  33  29  28  27  24  23  22  14  13  13  10  7  95 |
| Pharmacist | 544 |
| Nurse | 153 |
| Others (Medical Students etc.) | 175 |

From September 2018 to February 2020

SAMPOP = Saga application for management of drug holidays in perioperative periods, SUH = Saga university hospital
